# The Significance of Histopathologic Assessment in Bone Marrow Disease in Neuroblastoma

**DOI:** 10.5146/tjpath.2021.01556

**Published:** 2022-05-19

**Authors:** Sumeyye Ekmekci, Dilek Ince, Nur Olgun, Erdener Ozer

**Affiliations:** Department of Pathology, University of Health Sciences, Izmir Tepecik Research and Training Hospital, Izmir, Turkey; Department of Clinical Oncology, Dokuz Eylul University, Institute of Oncology, Izmir, Turkey; Department of Pathology, Dokuz Eylul University, School of Medicine, Izmir, Turkey

**Keywords:** Bone marrow biopsy, Neuroblastoma, Metastasis, Tumor differentiation, Tumor percentage

## Abstract

*
Objective:* Neuroblastoma (NB) is the most common extracranial solid tumor in children and is responsible for 12% of cancer-related deaths. The status of metastatic disease in the bone marrow (BM) is a predictor of poor outcome. The purpose of this study was to investigate the predictive significance of histopathological examination of BM in NB.

*
Material and Method:* The study included 61 cases with archival bone marrow biopsy tissues. The cases were evaluated regarding the percentage of metastatic tissue and its differentiation. Primary tumor slides were also reviewed to perform the Shimada classification based on the differentiation status and mitosis-karyorrhexis index. The patients’ age, gender, *NMYC* amplification, clinical risk group, and disease outcome were also noted.

*
Results:* Of the 61 cases, 17 had BM involvement. Of those, eight cases (47.1%) were refractory NB showing disease relapse. Based on BM examination, five cases (29.4%) were categorized as complete response, seven (41.2%) as progressive disease, three (17.6%) as minimal disease, and two (11.8%) as stable disease. The progressive disease category was significantly related with refractory disease and *NMYC* amplification along with the high-risk category (p =0.002 and p= 0.003 respectively). Undifferentiated histology and presence of more than 20% of tumor tissue in the BM biopsy at diagnosis were significantly associated with the progressive disease category (p=0.01 and p<0.001, respectively).

*
Conclusion:* We conclude that evaluating the percentage of metastatic tumor tissue and tumor differentiation in BM biopsies is of clinical importance in the management of neuroblastoma patients.

## INTRODUCTION

Neuroblastomas (NB) are the most common extracranial solid tumors in children less than 15 years of age (1–9). They are responsible for approximately 15% of cancer-related deaths in this age group (2–10). There are established parameters in the risk stratification of NB patients such as tumor differentiation and mitosis-karyorrhexis index (MKI), *NMYC* amplification, the patient’s age, and tumor stage (11). According to the International Neuroblastoma Risk Group (INRG) staging system, metastasis at the time of diagnosis is considered a significant indicator of poor prognosis (1). If the tumor has spread to distant sites such as the lymph nodes, bone, liver, skin, bone marrow (BM), or other organs, it meets the criteria for stage 4. BM is the most common metastatic site in NB patients at the time of diagnosis and one of the common localizations of disease recurrence (1,3,4,6,9,12–14).

In the past few decades, a significant improvement has been observed in the prognosis of certain well-defined NB patient subsets, whereas only a modest improvement has been reported in the prognosis of children of the high-risk groups (15). The presence of tumor tissue in the BM corresponds to not only advanced stage but also a high-risk category. NB patients with bone marrow involvement carry a high risk of refractory disease and are likely to show a poor prognosis. Therefore BM aspiration and biopsy are quick, easy and cost-effective methods and have become routine standard procedures to evaluate the prognosis in newly diagnosed patients and assess disease response without having to wait for greater tumor burden to develop (1,10). The information achieved by bilateral trephine BM biopsy interpretation is also valuable for the clinical follow-up strategy (1). We aimed in the present study to address the clinical importance of BM involvement in the management of NB patients, and also to test the significance of established histopathological criteria in predicting the prognosis.

## MATERIALS and METHODS

**Figure 1:**
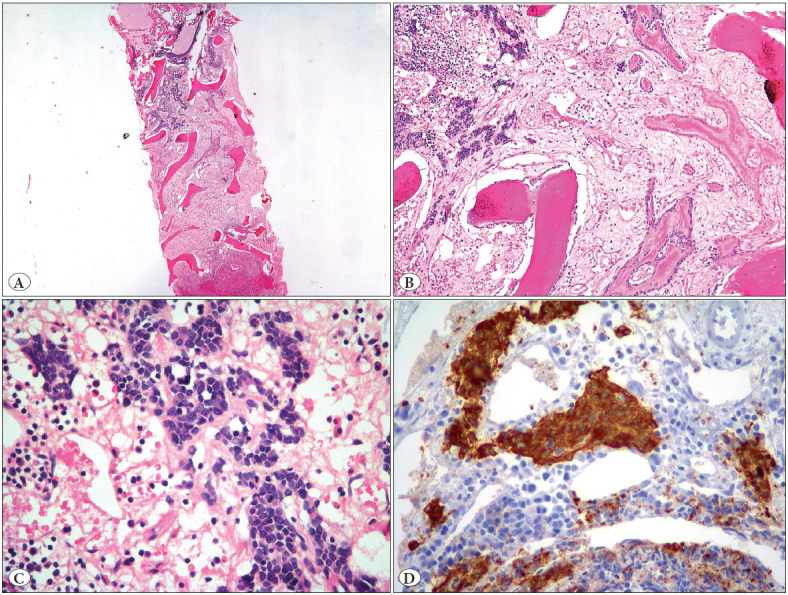
Neuroblastoma metastasis in bone marrow biopsy (involvement ratio: 15%). H&E, A x40, B x100, C x400, D: synaptophysin immunohistochemistry x400.

**Figure 2:**
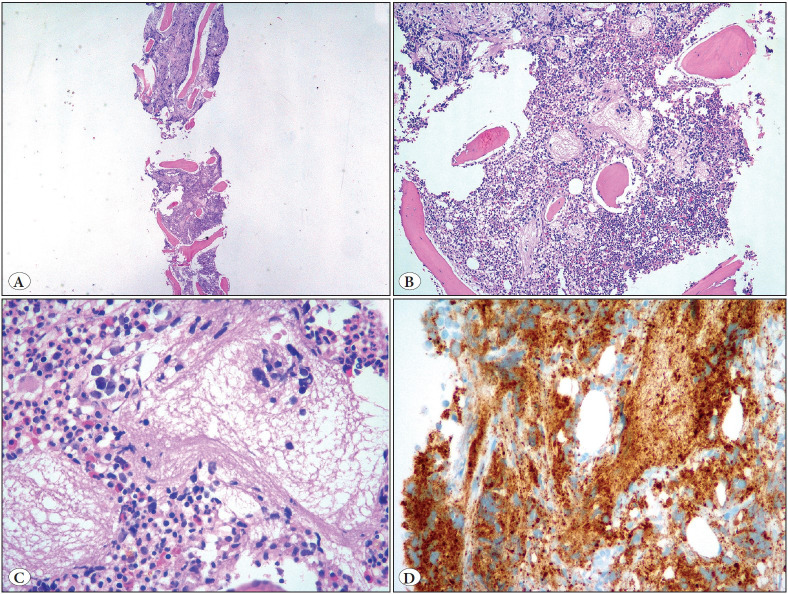
Neuroblastoma infiltration in bone marrow biopsy (involvement ratio: 70%). H & E; A x40, B x100, C x400, D: synaptophysin immunohistochemistry, x400.

The study initially included 86 NB cases diagnosed and treated at our institution. Of those, 25 cases were excluded because archival bilateral BM trephine biopsy materials were unavailable. Histological slides of the remaining 61 patients were reviewed and tumor involvement at diagnosis and during follow-ups was confirmed in 17 cases. These cases were categorized according to the percentage of metastatic tumor by two expert pathologists (EO, SE), as proposed in the literature (1,16). Additional sections of hematoxylin and eosin (H&E)-stained and immunostained slides with at least three antibodies (synaptophysin, chromogranin and PGP 9.5) were used to determine the amount of tumor (1,17,18). The surface area involved by the neuroblastic tumor was given as a percentage in the BM tissue. Based on the percentage of tumor tissue in BM biopsy at diagnosis and repeated biopsies during follow-ups, the disease status of the cases was categorized according to the criteria in Table 1. The differentiation status of the tumor in BM at diagnosis was grouped as well-differentiated, poorly-differentiated and undifferentiated, along with the percentage of the tumor tissue as <5%, 5-20% and >20% (Figure 1A-D, Figure 2A-D).

**Table 1:** The categorization of response status based on bone marrow involvement.

**Status**	**Criterion**
Complete Response	No tumor involvement in the follow-up biopsy
Minimal Disease	Any of the following:
	<5% involvement in the follow-up biopsy of a non-metastatic tumor at diagnosis Involvement in the follow-up biopsy remains same, while it is < 5% involvement at diagnosis. Involvement decreased to <5% in the follow-up biopsy, while it is > 20% at diagnosis.
Stable Disease	Involvement is > 5% in the follow-up biopsy, but the criteria did not meet any of the above groups.
Progressive Disease	> 5% involvement in the follow-up biopsy in a non-metastatic tumor at diagnosis, OR increased to two times tumor tissue in the follow-up biopsy than at diagnosis and there is > 20% involvement

Archival histological slides of the primary tumor tissues of 17 patients were also reviewed by two pathologists (EO, SE) to assess the Shimada category based on tumor differentiation and MKI. Demographic data such as patients’ age and gender, clinical data including tumor *NMYC* amplification status, stage, patient risk group and refractory status were obtained from the patient records.

Clinical and demographic characteristics of the patients were defined by using descriptive statistics. The statistical relationship of both BM metastatic tumor percentage category and differentiation with clinical and demographic characteristics was evaluated with the chi-square test. A probability level of 0.05 or less was chosen to represent statistical significance. All p-values were two-sided and denoted by *p*. Fisher’s exact test was used to calculate *p* values, as the cell frequencies were too small for the standard chi-squared test to be accurate.

Approval for the study was granted by the Local Medical Ethics Committee (approval No. 2021/5-2). All study procedures were performed according to the Declaration of Helsinki principles.

## RESULTS

A total of 86 neuroblastoma cases were examined for the study, and 61 cases had BM data. Of the 61 cases, 17 had BM involvement. Eleven of these cases were boys, and six were girls. Nine patients were older than 5 years old, one younger than 18 months, and seven between 18 months and 5 years old. Sixteen (54.1%) cases were stage IV and one case (5.9%) was stage IVS. Eleven patients (64.7%) were in the high-risk category and six (35.3%) in the intermediate risk group. *MYCN *amplification was detected in 11 (64.7%) cases. The median follow-up was 28 months. Refractory disease was observed in eight (47.1%) of the cases.

Histologically, four tumors were undifferentiated NBs, and 13 were poorly differentiated. Four cases had low, one case had moderate MKI and 12 cases had high MKI. All tumors had unfavorable Shimada histology. Of the tumors with BM involvement, five (29.4%) were undifferentiated NBs and 12 (73.6%) were poorly differentiated. There were eight cases (47.1%) with BM involvement less than 5%, three (17.6%) with 5-20%, and six (35.3%) with more than 20%. Based on BM involvement criteria, seven cases (41.2%) were progressive disease, five (29.4%) complete response, three (17.6%) minimal disease, and two (11.8%) stable disease.

Table 2 demonstrates the statistical relationship between BM disease status and the prognostic parameters. There was no statistically significant relationship between disease status and gender, age, tumor differentiation and MKI. A statistically significant relationship was found between progressive disease and BM involvement percentage more than 20% (p<0.001), lack of differentiation of the tumor (p=0.001), relapse (p=0.002), high-risk category (p=0.003), and *N-MYC* amplification (p=0.003). Progressive disease and *NMYC *amplification were significantly related with refractory disease (p=0.002 and p=0.003 respectively).

**Table 2:** The relation between bone marrow status and clinicopathological characteristics of the study group.

**Variable**	**n (%)**	**Complete response** **n=5 (29.4%)**	Progressive disease* **n=7 (41.2%)**	**Minimal disease** **n=3 (17.6%)**	**Stable disease** **n=2 (11.8%)**
**Gender**					
Male	11 (64.7)	4	4	2	1
Female	6 (35.3)	1	3	1	1
**Histologic type**					
Poorly Differentiated	13 (76.5)	4	4	3	2
Undifferentiated	4 (23.5)	1	3	0	0
**MKI**					
Low	4 (23.5)	1	2	0	1
Moderate	1 (5.9)	1	0	0	0
High	12 (73.6)	3	5	3	1
**Age**					
<18 months	1 (5.9)	1	0	0	0
18 months –5 years	7 (41.2)	4	2	0	1
>5 years	9 (52.9)	0	5	3	1
***NMYC*** **amplification**					
Negative	6 (35.3)	5	0	1	0
Positive*	11 (64.7)	0	7	2	2
**Risk group**					
Intermediate	6 (35.3)	5	0	1	0
High *	11 (64.7)	0	7	2	2
**Refractory disease**					
No	9 (52.8)	5	0	3	1
Yes*	8 (47.1)	0	7	0	1
**Tumor differentiation**					
Undifferentiated *	5 (29.4)	0	5	0	0
Poorly differentiated	12 (73.6)	5	2	3	2
**Bone marrow involvement rate**					
<5%	8 (47.1)	5	0	3	0
5-20%	3 (17.6)	0	1	0	2
>20%*	6 (35.3)	0	6	0	0

* Indicates statistical significance, **MKI:** Mitosis-karyorrhexis index

## DISCUSSION

In the evaluation of NBs, the International Neuroblastoma Staging System (INSS) defines these tumors by resectability and tumor location in relation to the presence or absence of regional or distant metastatic spread (17,19,20). According to this definition, the stage of the disease, the age of the patient and the tumor biological factors form the basis of the therapeutic risk classification (19). When the case distribution in the risk classification of NB is taken account, about half of the cases have a high-risk phenotype and a low survival. Five-year survival is 30-40% in the high-risk group, whereas NBs in the low or intermediate risk group have excellent survival (21).

Almost half of NBs have metastases at the time of diagnosis (3,17). Although the most common localization of metastases is the BM, metastases can be observed in the skeleton, lymph nodes, liver, and intracranial and orbital regions (3,4,6,7,17,21,22). Despite aggressive multimodality therapy, the long-term survival rate at diagnosis of metastatic disease for patients older than 18 months is still less than 30% (15).

BM is the major site of metastasis in advanced neuroblastoma, and detection of even minimal residual neuroblastoma cells in this region is associated with a poor prognosis (9,17). However, the potential for metastatic spread may not be equal among all patients with NB (19). Although it is a well-known tumor with its different behaviors and the connections between these behaviors and molecular patterns, the metastatic propagation mechanisms of NBs are not fully understood. Studies have shown that some tumors have a higher risk of metastatic spread than others as a result of searching the factors associated with metastatic spread and BM infiltration (19).

Since the determination of BM metastasis is a key point in evaluating the course of NB, core biopsies are frequently taken as part of routine bone marrow sampling in pediatric patients in many institutions. An advantage of this sampling is that it allows the use of immunohistochemistry on these samples for detection of tumor cells (17). Previous studies have shown that immunohistochemical analysis is superior to routine H&E assessment (17) and it is recommended to use at least three immunohistochemical markers such as synaptophysin, chromogranin, PGP 9.5 and cyclin-D1 to demonstrate tumor tissue (1,13,17). Moreover, neuroblastic differentiation and the percentage of tumor tissue should be stated in the pathology report (1).

Amplification of the *MYCN* gene is observed in approximately 20-35% of all NBs and is an important molecular marker for identifying high-risk patients (3,4,14). The combination of *MYCN* overexpression and caspase-8 deletion significantly increases BM metastasis (3,4). However, it has been reported that BM metastasis is associated with poor prognosis in patients older than 1 year, regardless of *MYCN* amplification (10,13). In addition, BM status beyond other known predictive factors such as *MYCN* copy number was shown to provide independent prognostic information for patients over the age of 1 and with stage 4 NBs (18). In a study evaluating the *MYCN* status by using FISH and amplification, BM involvement was reported in 6% of the cases. In this study, all cases were high-risk and stage 4, and the criterion for positive BM involvement was the presence of neuroblastoma in at least 20% of the biopsy (14).

In the present study, the majority of the cases were in the high-risk group older than 18 months. *MYCN* amplification was observed in 70% of the cases with BM metastasis. In a study by Jo et al. (8), similar results were reported. Kuroda et al. (13) investigated the presence of circulating tumor cells (CTC) in the peripheral blood and/or BM micrometastases during treatment in advanced stage NB cases receiving chemotherapy. *MYCN* amplification was detected in 12 of these cases. However, neither CTC nor persistent BM micrometastases were associated with *MYCN* amplification. In our study, a statistically significant relationship between progressive disease based on BM involvement and *NMYC* amplification was found.

In our study, BM involvement over 20% and undifferentiated histology were related to refractory disease. In a study by Russell et al. (19), low-stage NB was unlikely to have metastatic disease in the BM, and therefore they recommended further investigation of the genetic factors such as *MYCN* amplification and chromosome 1p deletion in order to better predict which tumors are at risk of metastatic spread. In contrast, the presence of permanent BM micrometastasis after chemotherapy was reported as a predictor of poor prognosis (13). In addition *MYCN* amplification should be evaluated both in the primary tumor and BM metastases although the tumor may show genetic heterogeneity. It should be kept in mind that genetic differences that can be found in the primary tumor, and the metastatic tumor may also affect the treatment process (23).

In the study by Tian et al. (10), the presence of BM metastasis was seen in 75% of the cases at the time of diagnosis and 45% of the cases after treatment. Five-year event-free survival was found to be statistically different in patients with residual metastatic disease compared to patients without (15). In our study, a statistically significant relationship was found between the presence of more than 20% of tumor tissue in the BM biopsy at diagnosis and progressive disease. In conclusion, BM metastasis status at diagnosis and during follow-up appears to be a significant prognostic factor apart along with poor prognostic factors. The crucial cut-off point for BM involvement is 20% and a value over this ratio is significantly related to refractory disease. Our study demonstrates the prognostic and predictive significance of determining the tumor percentage and differentiation of NBs in the BM biopsies at diagnosis and during follow-up.

## Conflict of Interest

The authors have no conflicts of interest to declare.

## Funding

The authors have declared that they did not receive any financial support for this study.
